# 2021 ACC/AHA/SCAI Coronary Artery Revascularization Guidelines for Managing the Nonculprit Artery in STEMI

**DOI:** 10.1016/j.jaccas.2022.02.003

**Published:** 2022-04-06

**Authors:** Creighton W. Don, Brittany A. Zwischenberger, Paul A. Kurlansky, Sunil V. Rao, Garima Sharma, Jennifer S. Lawton, Jacqueline E. Tamis-Holland

**Affiliations:** a Department of Medicine, Division of Cardiology, University of Washington, Seattle, Washington, USA; b Division of Cardiothoracic Surgery, Duke University, Durham, North Carolina, USA; c Division of Cardiac Surgery, Columbia University, New York, New York, USA; d Department of Medicine, Division of Cardiology, Duke University, Durham, North Carolina, USA; e Ciccarone Center for the Prevention of Cardiovascular Disease, Division of Cardiology, Johns Hopkins School of Medicine, Baltimore, Maryland USA; f Department of Surgery, Division of Cardiac Surgery, Johns Hopkins School of Medicine, Baltimore, Maryland, USA; g Mount Morningside Hospital and the Icahn School of Medicine at Mount Sinai, New York, New York, USA

**Keywords:** complete revascularization, surgery, multivessel, myocardial infarction, nonculprit, noninfarct, percutaneous coronary intervention, STEMI, ST-segment elevation myocardial infarction, CABG, coronary artery bypass grafting, LAD, left anterior descending, LOE, level of evidence, OM, obtuse marginal, PCI, percutaneous coronary intervention, RCA, right coronary artery, STEMI, ST-segment elevation myocardial infarction

## Abstract

The 2021 Coronary Artery Disease revascularization guidelines of the American College of Cardiology (ACC), the American Heart Association (AHA), and the Society for Cardiovascular Angiography and Interventions (SCAI) provide recommendations for managing nonculprit arteries in ST-segment elevation myocardial infarction (STEMI). Although staged revascularization is preferred, at times same-setting intervention, coronary artery bypass surgery, or medical therapy may be preferable. These cases exemplify clinical scenarios for treating nonculprit arteries in STEMI. (**Level of Difficulty: Intermediate**.)

## Central Illustration

**Figure:**
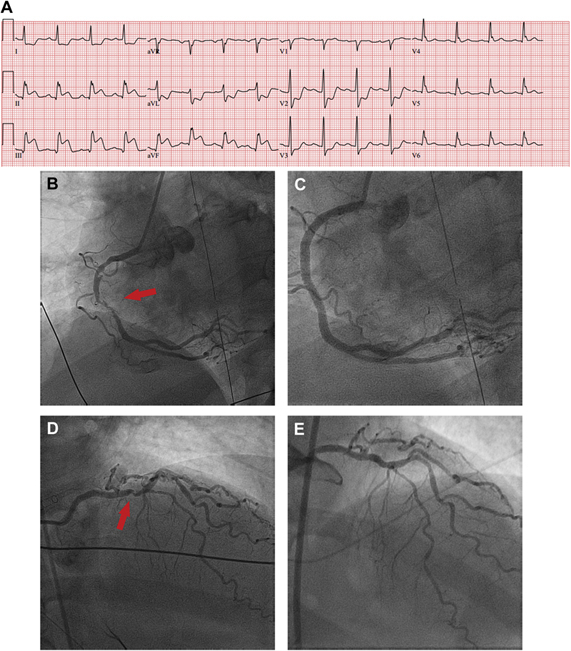

The management of multivessel coronary artery disease in patients with ST-segment elevation myocardial infarction (STEMI) who have received successful treatment of a culprit artery has evolved considerably over the past decade. The results of randomized trials^1^, ^2^, ^3^, ^4^, ^5^, ^6^ have had an impact on the approach advocated by the 2021 coronary artery revascularization guidelines of the American College of Cardiology (ACC), the American Heart Association (AHA), and the Society for Cardiovascular Angiography and Interventions (SCAI) (Table 1).^7^ In meta-analyses, incorporating the recently published COMPLETE trial (Complete versus Culprit-Only Revascularization Strategies to Treat Multivessel Disease after Early PCI for STEMI), which randomized >4,000 patients,^1^ multivessel revascularization reduced major adverse cardiovascular events in comparison with percutaneous coronary intervention (PCI) of only the culprit artery.^8^^,^^9^ The benefit of multivessel revascularization in specific clinical situations (e.g., patients with shock or renal insufficiency)^2^ or the timing of the revascularization (same setting versus staged), however, is less clear.

**Learning Objectives**

• Identify patients appropriate for revascularization (PCI or surgery) of nonculprit arteries in patients with STEMI, based on 2021 AHA/ACC/SCAI revascularization guidelines.
• Define clinical and anatomic conditions that would favor same-setting versus staged nonculprit revascularization

**Table 1:** 2021 ACC/AHA/SCAI Coronary Artery Revascularization Guideline 5.2 for Revascularization of the Noninfarct Artery in Patients With STEMI

COR	LOE	Recommendations
1	A	1. In selected patients in hemodynamically stable condition with STEMI and multivessel disease, after successful primary PCI, staged PCI of a significant noninfarct artery stenosis is recommended to reduce the risk of death or MI.^1^, ^2^, ^3^, ^4^, ^5^, ^6^
2a	C-EO	In selected patients with STEMI with complex multivessel noninfarct artery disease, after successful primary PCI, elective CABG is reasonable to reduce the risk of cardiac events.
2b	B-R	In selected patients in hemodynamically stable condition with STEMI and low-complexity multivessel disease, PCI of a noninfarct artery stenosis may be considered at the time of primary PCI to reduce cardiac event rates.^3^^,^^4^^,^^6^
3: Harm	B-R	In patients with STEMI complicated by cardiogenic shock, routine PCI of a noninfarct artery at the time of primary PCI should not be performed because of the higher risk of death or renal failure.^2^

ACC = American College of Cardiology; AHA = American Heart Association; B-R = B-randomized; CABG = coronary artery bypass grafting; C-EO = C-expert opinion; COR = class of recommendation; LOE = level of evidence; MI = myocardial infarction; PCI = percutaneous coronary intervention; SCAI = Society for Cardiovascular Angiography and Interventions; STEMI-ST = elevation myocardial infarction.

Reprinted from Lawton et al.^7^

We present 3 cases illustrating the challenges of treating patients with STEMI and multivessel disease, highlighting how the new recommendations guide clinical decision making, and we illustrate situations that may not fall precisely within the guidelines, where clinical judgment and heart team discussions remain critical.

## Case 1: Nonculprit Arteries and Staged Pci

**Figure 1:**
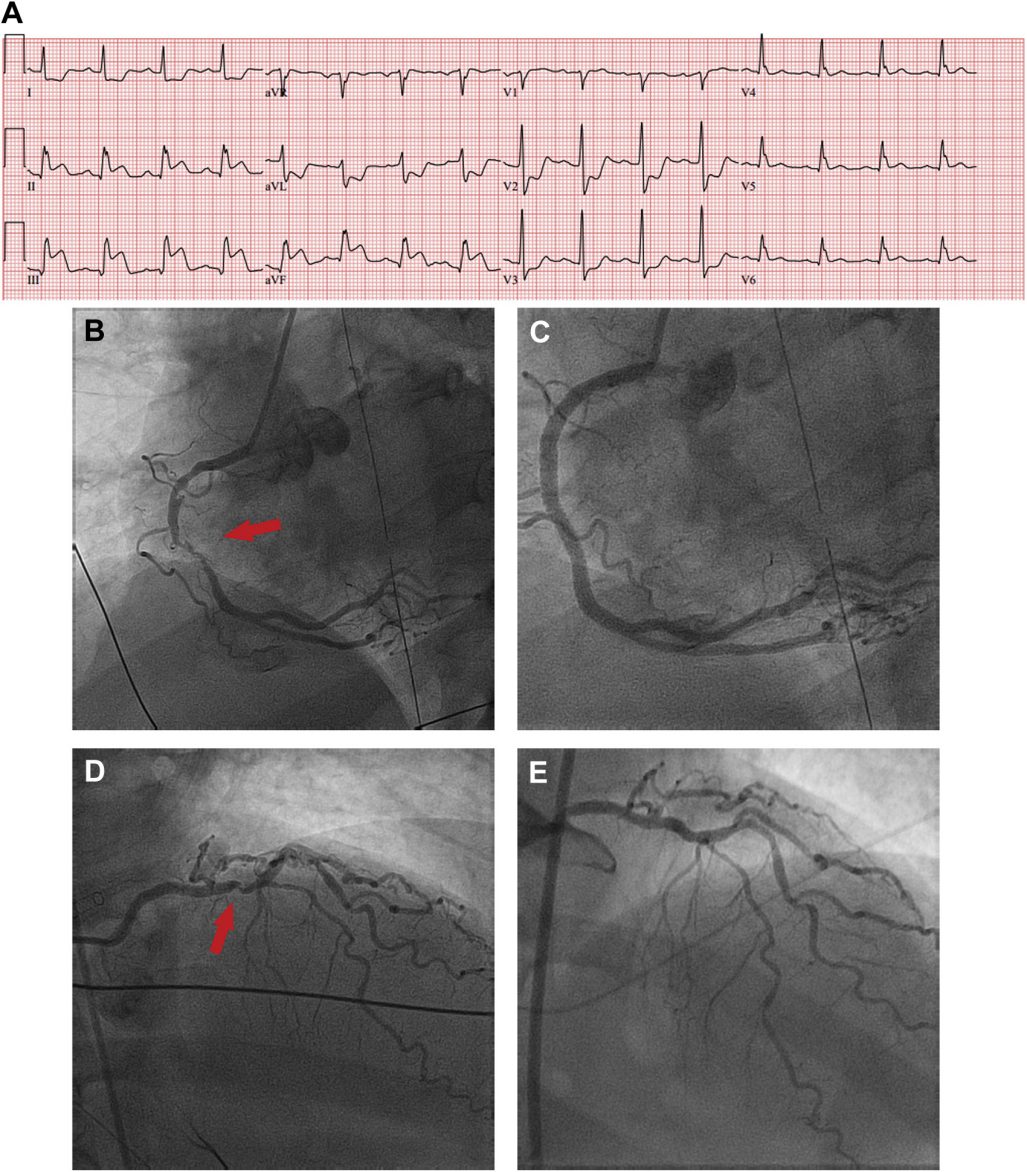
Staged Revascularization of a Nonculprit Artery **(A)** Inferior STEMI—RCA culprit artery. **(B)**. Mid-RCA 80% ulcerated culprit lesion. **(C)** Post–mid-RCA stenting. **(D)** Baseline 70% mid-LAD nonculprit lesion. **(E)** Staged stenting of the mid-LAD proximal to the D1 bifurcation.

A 48-year-old woman with systemic lupus erythematous, tobacco abuse, and hypertension presented with an inferior STEMI (Figure 1A) complicated by ventricular fibrillation, with blood pressure (BP) 80 mm Hg by palpitation and intermittent second-degree heart block, with heart rate 40 beats/min.

Angiography demonstrated tandem ulcerated and distal hazy 80% stenoses in the mid–right coronary artery (RCA) (Figure 1B) and an eccentric ulcerated 80% mid–left anterior descending (LAD) stenosis with a small, diffusely diseased LAD (Figure 1C).

A temporary pacing wire was placed. The mid-RCA lesion was thrombotic with recanalization and was stented (Figure 1B). The patient’s hypotension and bradycardia resolved.

An ischemia-guided approach to evaluate the diffusely diseased LAD was planned, but on the third day after STEMI she experienced an episode of typical chest pain with nonspecific T-wave changes on electrocardiogram. She underwent PCI of the eccentric ulcerated 80% mid-LAD stenosis; the distal LAD was very small, with only moderate atherosclerosis on intravascular ultrasound, and did not undergo intervention (Figure 1C). The patient’s angina resolved, and she is free from cardiovascular events for the past 2 years.

This case illustrates a successful staged PCI of a nonculprit artery. The 2021 ACC/AHA/SCAI revascularization guidelines provide a class 1, level of evidence (LOA) A (“is recommended to reduce the risk of death or myocardial infarction) for staged PCI.^7^ In this case, staged PCI was initially deferred owing to lesion complexity and the patient’s initial hemodynamic instability.

## Case 2: Nonculprit Arteries and Cardiogenic Shock

**Figure 2:**
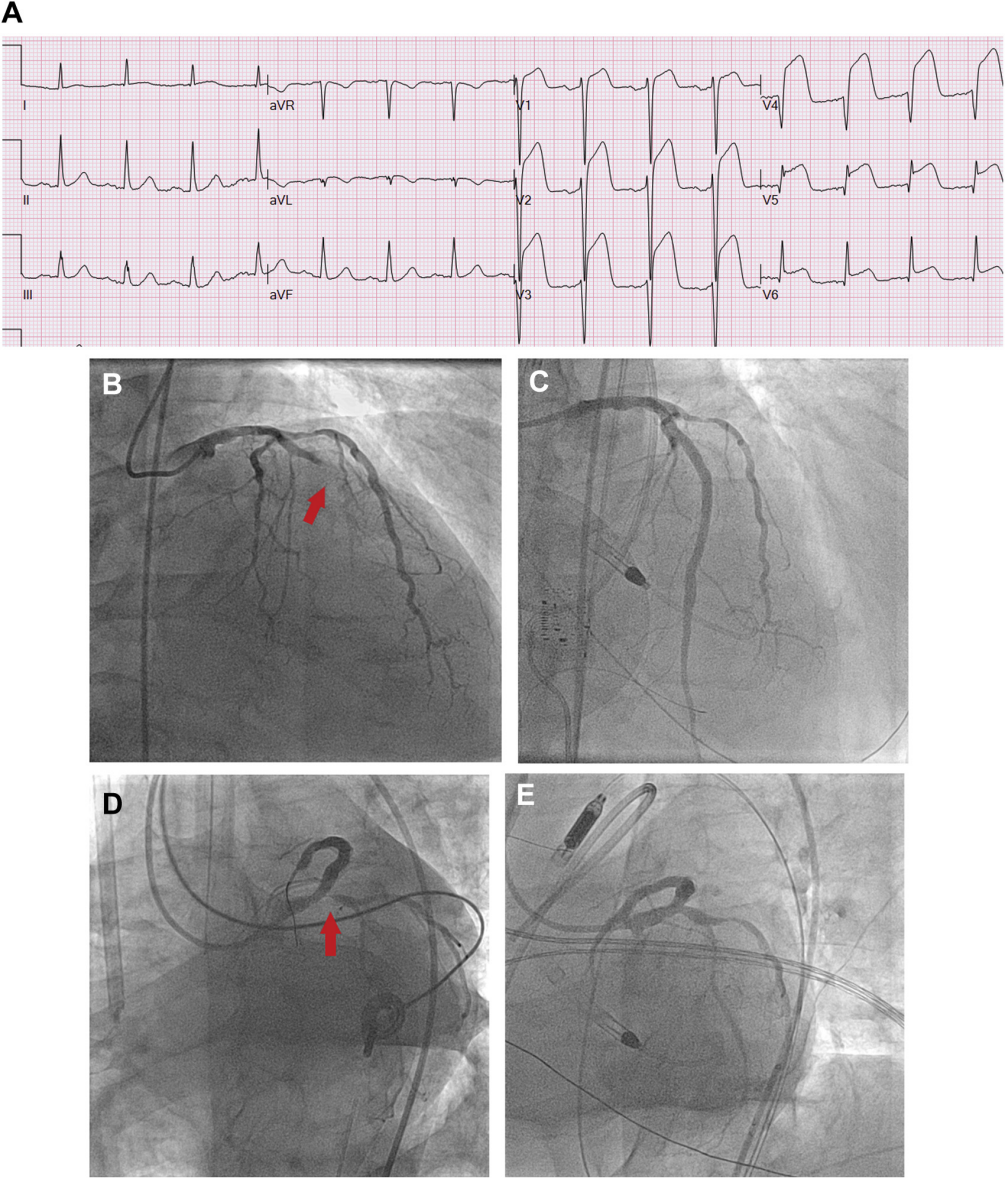

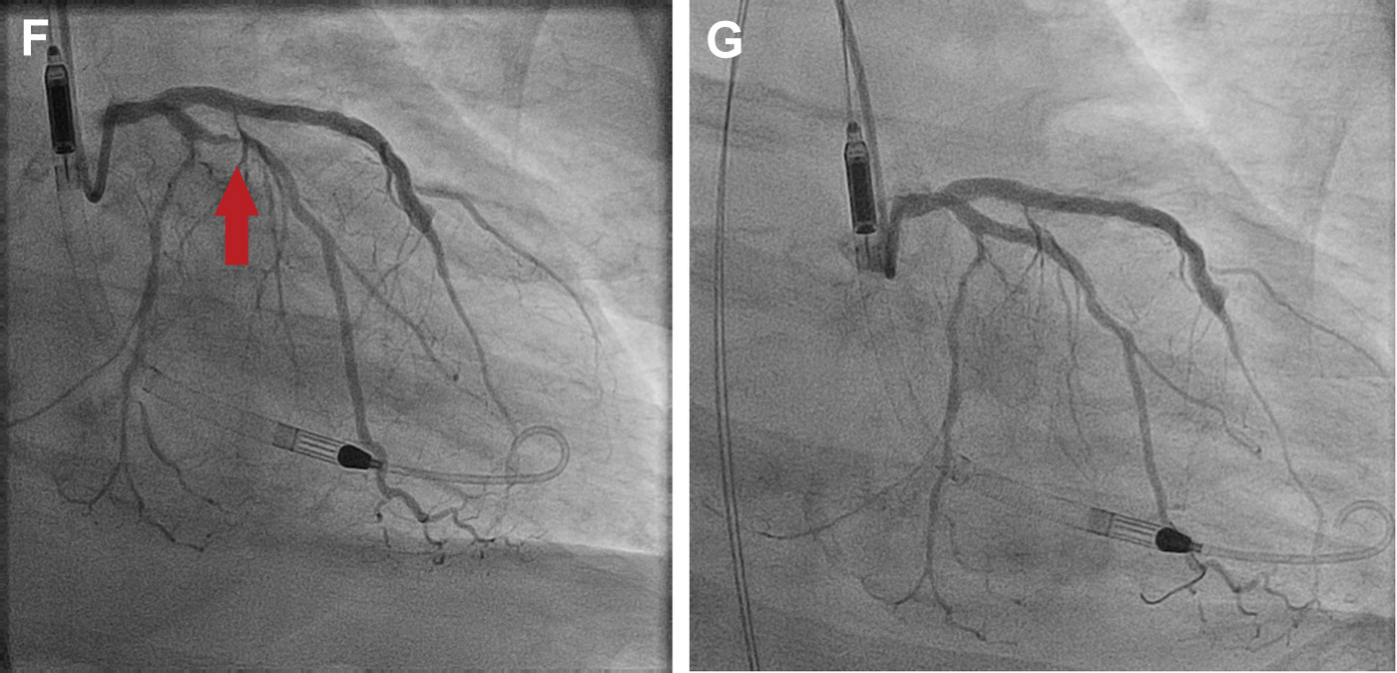
Revascularization of a Nonculprit Artery in a Patient With Cardiogenic Shock **(A)** Anterior STEMI—LAD culprit artery. **(B)** Mid-LAD 100% occlusion. **(C)** LAD after mid-LAD stenting. **(D)** Ostial circumflex 90% nonculprit lesion. **(E)** Same-setting stenting left main into circumflex. **(F)** Recurrent angina and 70% OM1 stenosis. **(G)** Staged stenting of nonculprit OM1 artery.

A 74-year-old man with diabetes, hypertension, prior PCI, renal cancer resection, and renal failure presented with ongoing chest pain for several weeks and an anterior STEMI (Figure 2A).

Angiography demonstrated an occluded LAD (Figure 2B). There was a moderate 60% proximal RCA stenosis, a 30% left main lesion involving an 80% ostial circumflex stenosis, and a 60% proximal stenosis in the first obtuse marginal (OM) (Figure 2C).

Stenting of the mid and proximal LAD was performed. Immediately after PCI, the patient experienced no-reflow in the distal LAD (Video 1), causing hemodynamic instability and ventricular fibrillation. He remained in severe cardiogenic shock despite defibrillation, cardiopulmonary resuscitation, vasopressors, and an intra-aortic balloon pump and subsequently an Impella (Abiomed) ventricular assist device. Despite the technically successful LAD PCI with subsequent TIMI flow grade 3 (Figure 2B, Video 2), the patient’s cardiogenic shock progressed (BP 65/39 mm Hg, heart rate 103 beats/min), requiring increasing ionotropic and vasopressor support. PCI of the circumflex as a same-setting procedure was thus used (Figure 2C), and the patient’s condition stabilized. The residual OM1 and RCA lesions were not treated immediately, but PCI of his OM1 was required before discharge (Figure 2D).

The patient was discharged free of chest pain but with an ejection fraction of 30% and is being treated for heart failure.

The 2021 ACC/AHA/SCAI coronary artery revascularization guidelines provide a class 3-harm, LOE-B-R recommendation (“should not be performed because of the higher risk of death or renal failure”) for routine multivessel PCI at the time of primary PCI in patients with STEMI complicated by cardiogenic shock.^7^ In context of the large circumflex territory contributing to severe refractory shock, however, PCI of this lesion was considered urgent in our clinical judgment and helped stabilize the patient’s condition.

## Case 3: Nonculprit Arteries and Coronary Artery Bypass Grafting

**Figure 3:**
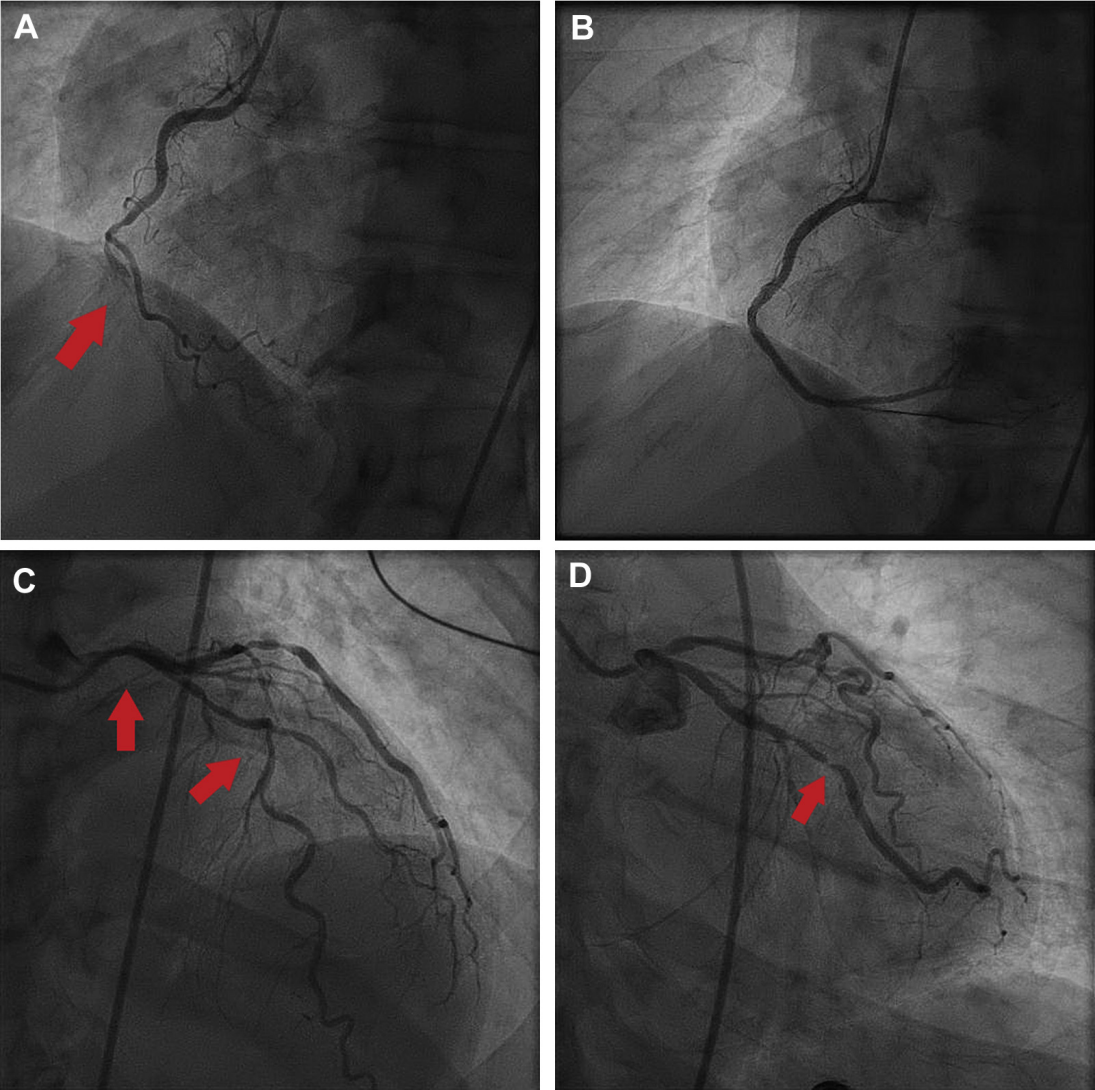
Coronary Artery Bypass Grafting for Treatment of Nonculprit Arteries **(A).** Occluded RCA culprit artery (100%). **(B)** Post-RCA stenting. **(C)** Left main 50% stenosis and diffuse LAD disease (nonculprit). **(D)** Obtuse marginal 90% nonculprit lesion.

A 66-year-old man presented with STEMI due to occlusion of the RCA (Figure 3A). Coronary angiography also demonstrated significant left main disease (60%), a mid-LAD (70%) stenosis, and OM (90%) disease (Figure 3B).

The RCA was successfully opened, but the patient experienced ventricular fibrillation, which was successfully defibrillated. He required a transvenous pacemaker for sinus bradycardia and an intra-aortic balloon pump for biventricular dysfunction. The patient’s condition stabilized, but revascularization of the remaining lesions was deferred because of cardiogenic shock (Class 3-harm, LOE-B-R). The patient did well after the procedure, with stabilization of hemodynamics. The patient was monitored to allow improvement in ventricular function, which normalized after 1 week. A heart team was convened for consideration of revascularization options. The patient was an excellent candidate for coronary artery bypass grafting (CABG), with a medical history of only hypertension. Because of the limited views of the left main, angiography was repeated confirming the presence of a significant left main stenosis, and the patient underwent CABG with the left internal mammary artery to the LAD and saphenous vein graft to the OM.

The patient recovered well from surgery and was discharged home. At his 2-year follow-up visit, the patient had normal ventricular function.

This case exemplifies the 2021 ACC/AHA/SCAI coronary artery revascularization guideline recommendation for CABG surgery to treat the nonculprit artery in patients with STEMI and complex disease (Class 2A, LOE-C-EO). Although the literature lacks a well-designed randomized controlled trial demonstrating the benefit of CABG for nonculprit arteries after STEMI, the role for CABG in this setting is inferred from studies of patients who have complexity coronary anatomy appropriate for surgical intervention (e.g., left main or high SYNTAX score).^7^

## Discussion

**Figure 4:**
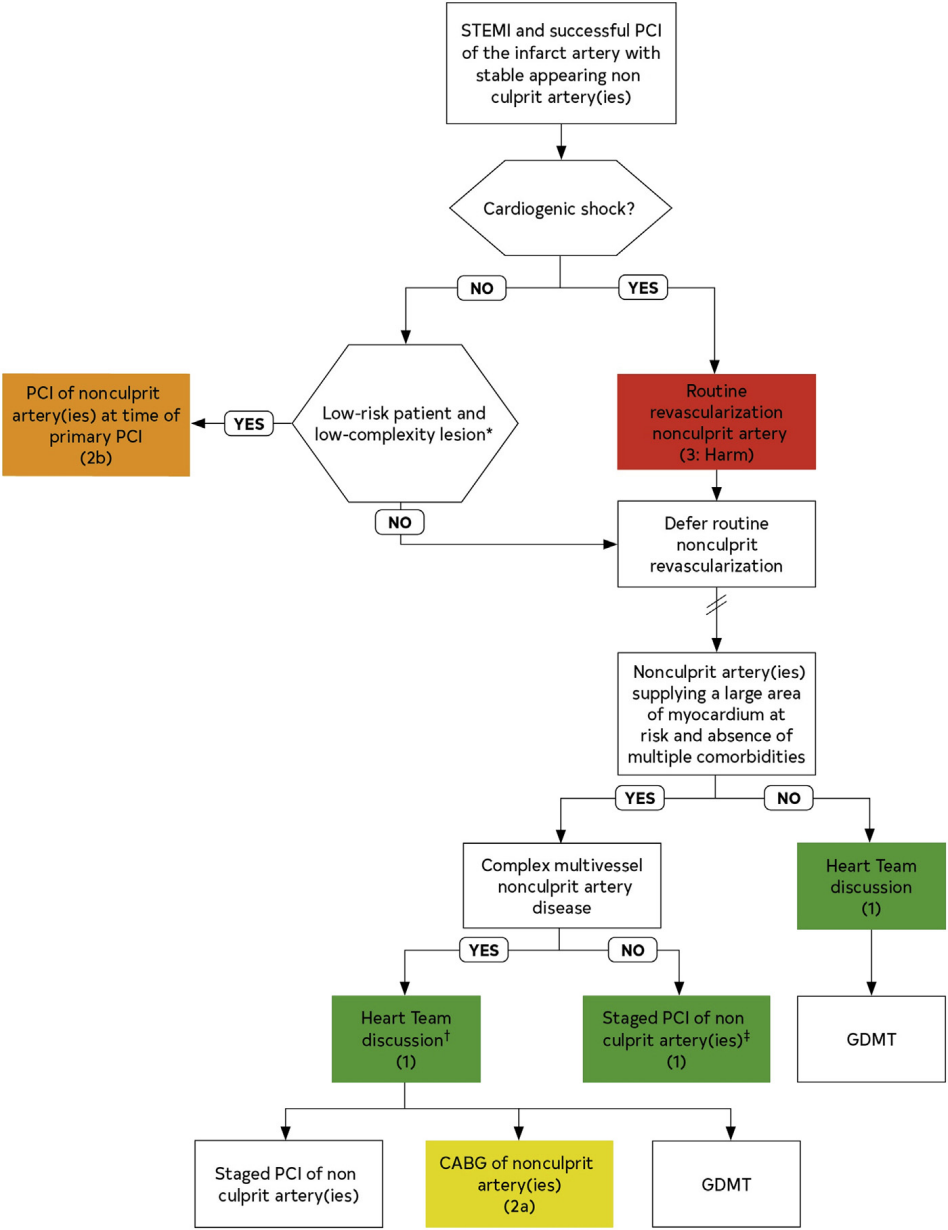
Revascularization of Nonculprit Coronary Artery Lesions in Patients With STEMI CABG = coronary artery bypass grafting; GDMT = guideline directed medical therapy; PCI = percutaneous coronary intervention; STEMI = ST-segment elevation myocardial infarction.

The 2021 ACC/AHA/SCAI revascularization guidelines provide detailed recommendations for treating patients with STEMI and multivessel disease, summarized by the treatment algorithm guiding the mode and timing of complete revascularization based on the anatomic/patient complexity and the presence of hemodynamic stability (Figure 4). Whereas the guideline recommendations help clinical decision making in many scenarios, our cases also illustrate the challenges in treating these patients and the limitations of the guidelines in some clinical situations.

The current recommendations advocate for staged PCI of nonculprit arteries to achieve complete revascularization in hemodynamically stable patients with STEMI after successful PCI of the culprit artery (Class 1, LOE-A).^7^ This is based on the COMPLETE trial, the largest randomized study of multivessel PCI in STEMI, showing a reduction in the combined risk of death or recurrent myocardial infarction with staged PCI of severe nonculprit arteries within 45 days of the index event.^1^ Several smaller trials of multivessel PCI in STEMI show a benefit to same-setting multivessel PCI in comparison with culprit-only PCI.^3^^,^^4^^,^^6^ In many of these studies, the benefit of multivessel PCI was driven by a reduction in repeated revascularization. For this reason, the guidelines state that same-setting multivessel PCI may be considered (Class 2B, LOE-B-R) in patients in stable condition with STEMI with low-complexity anatomy and without significant comorbidities.^7^

In case 2, PCI of the nonculprit left main and proximal circumflex was a high-risk intervention in a hemodynamically compromised patient; however, this stenosis contributed to the patient’s instability even though it was not the culprit artery. In complex patients, as in this patient, it is possible to have more than 1 culprit artery.

The use of CABG to treat the nonculprit arteries in STEMI has not been studied in clinical trials; however, given that CABG is favored over PCI in patients with complex coronary artery disease (section 8.1, Class 2A, LOE B-R “is reasonable”), the revascularization committee thought that the indication for CABG in a patient with STEMI and complex, nonculprit artery disease should involve a heart team approach and parallel the recommendations provided for patients in stable condition.^7^

## Take-Home Points

For patients with STEMI and successful PCI of the culprit arteries, the following treatment options for residual nonculprit coronary artery arteries are recommended:

• Staged multivessel revascularization for patients in stable condition
• Same-setting PCI may be considered for patients with low-risk anatomy without significant comorbidities
• Avoid nonculprit PCI in patients with cardiogenic shock unless there is clear evidence that such arteries are directly contributing to the hemodynamic instability
• CABG is reasonable in selected patients

## Funding Support and Author Disclosures

The authors have reported that they have no relationships relevant to the contents of this paper to disclose.

## Appendix

Supplemental Video 1 LAD no-reflow after PCI.

Supplemental Video 2 LAD after resuscitation.
